# Supplementary material for: Readable High-Speed Racetrack Memory Based on an Antiferromagnetically Coupled Soft/Hard Magnetic Bilayer
Source: Nanomaterials (Basel). 2019 Oct 30;9(11):1538. doi: 10.3390/nano9111538 (PMC6915474; doi:10.3390/nano9111538)
Supplement: Supplementary file 1 [file nanomaterials-09-01538-s001.pdf]

Supplementary Materials

Readable High-Speed Racetrack Memory Based on an Antiferromagnetically Coupled Soft/Hard Magnetic Bilayer

Ziyang Yu [1], Chenhuinan Wei [1], Fan Yi [2,*] and Rui Xiong [1]

[1] Key Laboratory of Artificial Micro- and Nano-structures of Ministry of Education, School of Physics and Technology, Wuhan University, Wuhan 430072, China

[2] School of Electronics and Information Engineering, Wuhan Donghu University, Wuhan 430212, China

* Correspondence: fyi@whu.edu.cn

S1. The deviation of Equation (1)–(4) in the main text through the cooperative coordinate method (CCM)

The normalized vector for the direction of magnetization is described as:

$$\overrightarrow{m_L} = (\sin\theta_L \cos\varphi_L, \sin\theta_L \sin\varphi_L, \cos\theta_L),$$

$$\overrightarrow{m_U} = (\sin\theta_U \cos\varphi_U, \sin\theta_U \sin\varphi_U, \cos\theta_U).$$

Here, the subscript *L* and *U* represent the lower and upper layers. The polar angle *θ* and the azimuthal angle *φ* are included in the ansatz for the DW magnetization for the lower and upper layer [1]:

$$\theta_L = 2\arctan\{\exp[(x-q_L)/\Delta_L]\},\ \varphi_L = \varphi_L(t) \tag{S1}$$

$$\theta_U = 2\arctan\{\exp[(x-q_U)/\Delta_L]\}+\pi,\ \varphi_U = \varphi_U(t) \tag{S2}$$

Here *t* is time, and *q* means the central position, and

$$\Delta_L = \sqrt{A_L/(K_L - \frac{1}{2}\mu_0 M_L^2)} \tag{S3}$$

$$\Delta_U = \sqrt{A_U/(K_U - \frac{1}{2}\mu_0 M_U^2)} \tag{S4}$$

are the width of the DWs for the lower and upper layers, respectively. Here *A*, μ_0, M_S, and *K* are the exchange stiffness constant, vacuum permeability, saturation magnetization, and uniaxial magnetic anisotropy constant for the PMA film, respectively.

The Thiele equation for the DW motion is derived via the Lagrangian approach. Let *l* be the Lagrangian density function of the bilayer system and it can be expressed as follow,

$$
\begin{aligned}
l &= E_{ex}^{L}+E_{a}^{L}+E_{d}^{L}+E_{DM}^{L}+E_{ex}^{U}+E_{a}^{U}+E_{d}^{U}+E_{DM}^{U}+\frac{M_L}{\gamma}\dot{\varphi}_L\cos\theta_L+\frac{M_U}{\gamma}\dot{\varphi}_U\cos\theta_U \\
&-\frac{J_{ex}}{\sqrt{(q_L-q_U)^2+t_s^2}}[\sin\theta_L\sin\theta_U\cos(\varphi_L-\varphi_U)+\cos\theta_L\cos\theta_U] \\
&=(\frac{A_L}{\Delta_L^2}+K_L-\frac{1}{2}\mu_0M_L^2+\frac{1}{2}\mu_0N_x^LM_L^2\cos^2\varphi_L)\sin^2\theta_L+\frac{D\sin\theta_L\cos\varphi_L}{\Delta_L}+\frac{M_L}{\gamma}\dot{\varphi}_L\cos\theta_L \\
&+(\frac{A_U}{\Delta_U^2}+K_U-\frac{1}{2}\mu_0M_U^2-\frac{1}{2}\mu_0N_x^UM_U^2\cos^2\varphi_U)\sin^2\theta_U+\frac{D\sin\theta_U\cos\varphi_U}{\Delta_U}+\frac{M_U}{\gamma}\dot{\varphi}_U\cos\theta_U \\
&-\frac{J_{ex}}{\sqrt{(q_L-q_U)^2+t_s^2}}[\sin\theta_L\sin\theta_U\cos(\varphi_L-\varphi_U)+\cos\theta_L\cos\theta_U]
\end{aligned}
\quad \text{(S5)}
$$

Here, E_{ex}, E_a, E_d, and E_{DM} represent the inter-layer exchange energy density, the uniaxial magnetic anisotropy energy density, the demagnetization energy density, and the DMI energy density. In the Lagrangian density function, $N_x^L=t_L\ln 2/(\pi\Delta_L), N_x^U=t_U\ln 2/(\pi\Delta_U)$ [2]. Here t_L, t_U, and t_s are the thickness of the lower layer, the upper layer, and the thickness of the NM layer, respectively.

The dissipation density function f_d should also be included to depict the dissipation of the bilayer system:

$$
\begin{aligned}
f_{\mathrm{d}} &= \sum_{i=L,U}(\alpha_iM_i/2\gamma)[d\vec{m}_i/dt-(\gamma_0/\alpha_i)H_{SO}^iJ(\vec{m}_i\times\vec{e}_y)]^2 \\
&=\sum_{i=L,U}\begin{array}{l}\frac{\alpha_iM_i}{2\gamma}\{[(\frac{-\dot{q}_i}{\Delta_i})^2+(\dot{\varphi}_i)^2]\sin^2\theta_i+(\frac{\gamma_0H_{so}^iJ}{\alpha_i})^2(1-\sin^2\theta_i+\sin^2\theta_i\cos^2\varphi_i) \\ +\frac{-2\gamma_0H_{so}^iJ}{\alpha_i\Delta_i}(\sin\theta_i\cos\varphi_i)\dot{q}_i+(\frac{-2\gamma_0H_{so}^iJ}{\alpha}\sin\theta_i\cos\theta_i\sin\varphi_i)\dot{\varphi}_i\}\end{array}
\end{aligned}
\quad \text{(S6)}
$$

Here α is the damping coefficient, and H_{SO} is the effective magnetic field of SOT as depicted in the Equation (7) in the main text.

The Lagrangian (L) and Rayleigh dissipation function (F) were determined by integrating l and f_d with respect to the entire space region for the track. The Thiele equations (Equation (1)–(4) in the main text) are finally deduced using the Lagrange–Rayleigh equation:

$$
\frac{\partial L}{\partial q_i}-\frac{d}{dt}(\frac{\partial L}{\partial\dot{q}_i})+\frac{\partial F}{\partial\dot{q}_i}=0 \quad \text{(S7)}
$$

S2. The duration time of the rising and falling edges as a function of magnetic parameters

Our simulation result indicates that the time for the rising edge is almost the same as that for the falling edge. As indicated in Figure S1, the time for the rising/falling edge can be reduced by increasing the damping coefficient, the DMI constant, and the interlayer exchange coupling, or by narrowing the gap of the uniaxial magnetic anisotropy constants of the two layers. This time can be smaller than 0.3 ns, which is much shorter than the period of the pulse in current electronic device. Therefore, the relaxation when the current is turned on or off does not influence the reading process significantly.

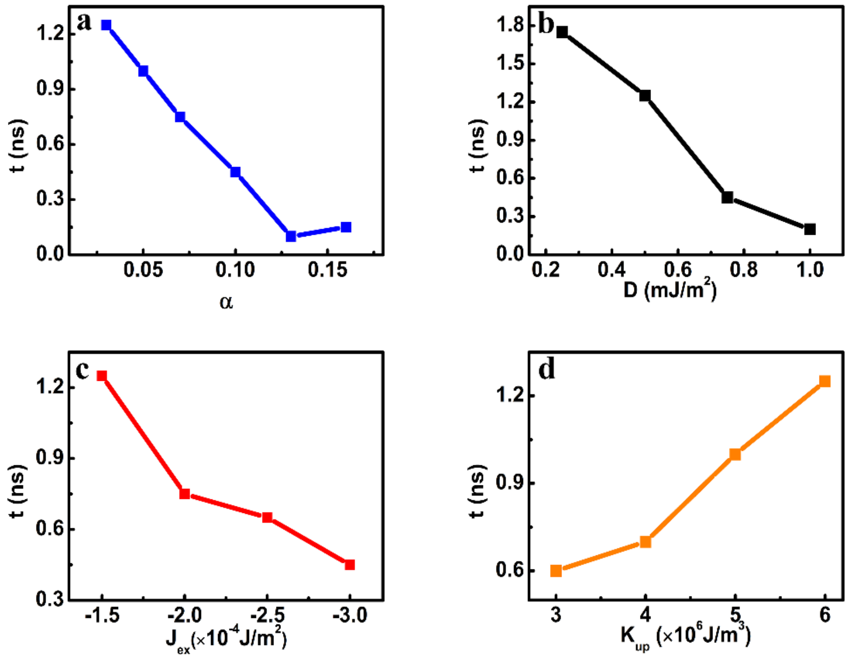

Figure S1. The variation of the time of the rising (falling) edges with different magnetic parameters, including (**a**) the damping factor ($J_{ex} = -2 \times 10^{-4}$ J/m², D = 0.75 mJ/m², $K_{up} = 6 \times 10^5$ J/m³, $K_{low} = 2 \times 10^5$ J/m³), (**b**) the DMI constant D ($J_{ex} = -2 \times 10^{-4}$ J/m², $\alpha = 0.1$, $K_{up} = 6 \times 10^5$ J/m³, $K_{low} = 2 \times 10^5$ J/m³), (**c**) the interlayer exchange coupling (J_{ex}) ($\alpha = 0.03$, D = 0.75 mJ/m², $K_{up} = 6 \times 10^5$ J/m³, $K_{low} = 2 \times 10^5$ J/m³), and (**d**) the uniaxial-anisotropy constant for the upper layer ($\alpha = 0.03$, D = 0.75 mJ/m², $J_{ex} = -2 \times 10^{-4}$ J/m², $K_{low} = 2 \times 10^5$ J/m³).

S3. The comparison of the DW motion induced by STT and SOT

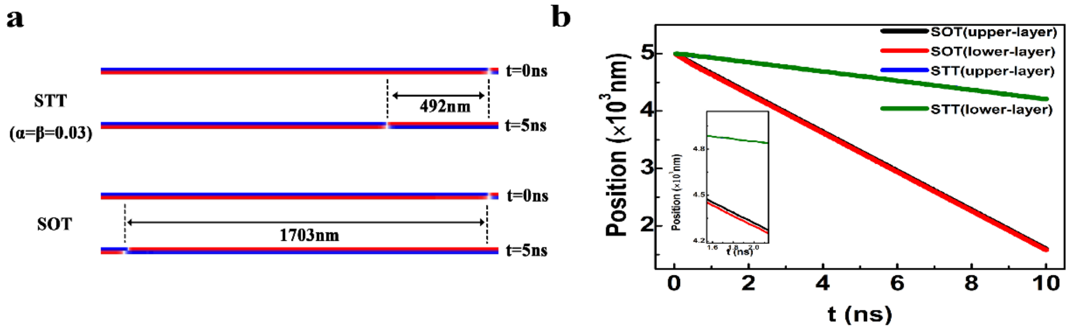

Figure S2. (**a**) The snapshots for the paired DWs driven by STT and SOT under the same current and other magnetic parameters, and (**b**) the temporal central positions of the upper and lower layers driven by STT and SOT ($J_{ex} = -2.5 \times 10^{-4}$ J/m², D = 0.75 mJ/m², $K_{up} = 6 \times 10^5$ J/m³, $K_{low} = 2.5 \times 10^5$ J/m³)

The motion of the paired DWs driven by SOT was compared with that by STT under the same current density and other parameters. In the STT case, we assume the nonadiabatic factor (β) equals the damping coefficient α. In this case, the DW may move very fast [3]. Our simulation results are shown in Figure S2 indicates that the velocity of DW driven by STT is obviously smaller than that by SOT under the same current density and other parameters. In addition, the staggered domain region was only observed in the SOT case. In a real multilayer,

the DW driven by STT can be even slower since the β is hard to estimate and the shunt of the current in different layers may be considered. In addition, it is also widely reported that the STT has a negligible contribution to the DW motion in the ultrathin HM/FM film [3–7]. Therefore, in the main text, we only consider the contribution from the SOT effect but neglect the STT effect.

References

[1]. Boulle, O.; Rohart, S.; Buda-Prejbeanu, L. D.; Jue, E.; Miron, I. M.; Pizzini, S.; Vogel, J.; Gaudin, G.; Thiaville, A., Domain wall tilting in the presence of the Dzyaloshinskii-Moriya interaction in out-of-plane magnetized magnetic nanotracks. Phys Rev Lett 2013, 111 (21), 217203.

[2]. Martinez, E.; Emori, S.; Perez, N.; Torres, L.; Beach, G. S. D., Current-driven dynamics of Dzyaloshinskii domain walls in the presence of in-plane fields: Full micromagnetic and one-dimensional analysis. Journal of Applied Physics 2014, 115 (21), 213909.

[3]. Yang, S. H.; Parkin, S., Novel domain wall dynamics in synthetic antiferromagnets. *Journal of Physics Condensed Matter* **2017,** *29* (30), 303001.

[4]. Emori, S.; Bauer, U.; Ahn, S. M.; Martinez, E.; Beach, G. S., Current-driven dynamics of chiral ferromagnetic domain walls. *Nature Materials* **2013,** *12* (7), 611-616.

[5]. Koyama, T.; Chiba, D.; Ueda, K.; Kondou, K.; Tanigawa, H.; Fukami, S.; Suzuki, T.; Ohshima, N.; Ishiwata, N.; Nakatani, Y.; Kobayashi, K.; Ono, T., Observation of the intrinsic pinning of a magnetic domain wall in a ferromagnetic nanowire. Nat Mater 2011, 10 (3), 194-7.

[6]. Cormier, M.; Mougin, A.; Ferré, J.; Thiaville, A.; Charpentier, N.; Piéchon, F.; Weil, R.; Baltz, V.; Rodmacq, B., Effect of electrical current pulses on domain walls in Pt/Co/Pt nanotracks with out-of-plane anisotropy: Spin transfer torque versus Joule heating. Physical Review B 2010, 81 (2).

[7]. Emori, S.; Beach, G. S., Roles of the magnetic field and electric current in thermally activated domain wall motion in a submicrometer magnetic strip with perpendicular magnetic anisotropy. J Phys Condens Matter 2012, 24 (2), 024214.
